# Supplementary material for: Personality and COMT gene: molecular-genetic and epigenetic associations with NEO-PI-R personality domains and facets in monozygotic twins
Source: Front Genet. 2024 Sep 25;15:1455872. doi: 10.3389/fgene.2024.1455872 (PMC11461223; doi:10.3389/fgene.2024.1455872)
Supplement: Supplementary file 1 [file Table1.DOCX]

**Personality and MB-COMT gene: molecular-genetic and epigenetic associations with NEO-PI-R personality domains and facets in monozygotic twins**

**Supplementary material**

Table A
Descriptive statistics by allele groups for the COMT gene on FFM personality domains and facets: molecular genetic sample

| Domain/Facet | COMT | M | SD |
| --- | --- | --- | --- |
| Neuroticism (domain) | Met+ | 86.67 | 15.90 |
|  | Val+ | 83.48 | 17.94 |
| Anxiety | Met+ | 16.54 | 3.42 |
|  | Val+ | 15.95 | 3.81 |
| Hostility | Met+ | 13.53 | 4.70 |
|  | Val+ | 12.84 | 4.91 |
| Depressiveness | Met+ | 13.23 | 4.06 |
|  | Val+ | 12.66 | 4.07 |
| Self-consciousness | Met+ | 14.99 | 4.17 |
|  | Val+ | 13.98 | 4.17 |
| Impulsivity | Met+ | 16.89 | 4.01 |
|  | Val+ | 17.27 | 4.60 |
| Vulnerability | Met+ | 11.50 | 4.27 |
|  | Val+ | 10.78 | 4.47 |
| Extraversion **(**domain**)** | Met+ | 114.19 | 19.69 |
|  | Val+ | 119.77 | 16.70 |
| Warmth | Met+ | 21.55 | 4.75 |
|  | Val+ | 23.22 | 4.33 |
| Gregariousness | Met+ | 19.57 | 4.82 |
|  | Val+ | 21.25 | 4.57 |
| Assertiveness | Met+ | 15.65 | 4.24 |
|  | Val+ | 15.82 | 4.07 |
| Activity | Met+ | 18.55 | 4.68 |
|  | Val+ | 19.38 | 4.39 |
| Excitement seeking | Met+ | 18.22 | 4.89 |
|  | Val+ | 18.37 | 5.25 |
| Positive emotions | Met+ | 20.65 | 3.87 |
|  | Val+ | 21.72 | 3.57 |
| Openness to experience (domain) | Met+ | 117.89 | 15.36 |
|  | Val+ | 117.42 | 14.83 |
| Fantasy | Met+ | 19.43 | 4.67 |
|  | Val+ | 19.06 | 4.41 |
| Aesthetics | Met+ | 21.44 | 4.19 |
|  | Val+ | 20.75 | 4.45 |
| Openness to feelings | Met+ | 21.60 | 3.58 |
|  | Val+ | 21.66 | 4.05 |
| Openness to action | Met+ | 16.92 | 3.66 |
|  | Val+ | 17.57 | 4.15 |
| Openness to ideas | Met+ | 19.39 | 4.96 |
|  | Val+ | 19.42 | 5.00 |
| Openness to values | Met+ | 19.11 | 3.38 |
|  | Val+ | 18.96 | 3.69 |
| Agreeableness (domain) | Met+ | 116.62 | 13.22 |
|  | Val+ | 118.90 | 13.57 |
| Trust | Met+ | 19.35 | 3.70 |
|  | Val+ | 19.89 | 3.64 |
| Straightforwardness | Met+ | 19.85 | 4.31 |
|  | Val+ | 20.54 | 3.46 |
| Altruism | Met+ | 22.58 | 3.54 |
|  | Val+ | 22.25 | 3.10 |
| Compliance | Met+ | 16.46 | 3.71 |
|  | Val+ | 17.03 | 3.62 |
| Modesty | Met+ | 17.06 | 4.88 |
|  | Val+ | 16.95 | 4.88 |
| Tender mindedness | Met+ | 21.34 | 4.08 |
|  | Val+ | 22.23 | 4.09 |
| Conscientiousness (domain) | Met+ | 124.88 | 17.25 |
|  | Val+ | 127.38 | 17.61 |
| Competence | Met+ | 21.91 | 3.53 |
|  | Val+ | 22.89 | 3.96 |
| Order | Met+ | 17.78 | 4.06 |
|  | Val+ | 17.40 | 4.10 |
| Dutifulness | Met+ | 24.71 | 3.57 |
|  | Val+ | 24.63 | 3.53 |
| Achievement striving | Met+ | 21.61 | 5.16 |
|  | Val+ | 22.84 | 4.29 |
| Self-discipline | Met+ | 20.72 | 4.44 |
|  | Val+ | 21.19 | 4.41 |
| Deliberation | Met+ | 18.16 | 5.06 |
|  | Val+ | 18.44 | 5.41 |

Table B

Correlations between NEO-PI-R domains and facets

|  | **1** | 2 | 3 | 4 | 5 | 6 | 7 | **8** | 9 | 10 | 11 | 12 | 13 | 14 | **15** | 16 | 17 | 18 |
| --- | --- | --- | --- | --- | --- | --- | --- | --- | --- | --- | --- | --- | --- | --- | --- | --- | --- | --- |
| **Neuroticism (domain - 1)** | 1 |  |  |  |  |  |  |  |  |  |  |  |  |  |  |  |  |  |
| Anxiety (2) | .607^**^ | 1 |  |  |  |  |  |  |  |  |  |  |  |  |  |  |  |  |
| Hostility (3) | .613^**^ | .294^*^ | 1 |  |  |  |  |  |  |  |  |  |  |  |  |  |  |  |
| Depression (4) | .696^**^ | .449^**^ | .179 | 1 |  |  |  |  |  |  |  |  |  |  |  |  |  |  |
| Self-conscientiousness (5) | .654^**^ | .264^*^ | .292^*^ | .439^**^ | 1 |  |  |  |  |  |  |  |  |  |  |  |  |  |
| Impulsiveness (6) | .666^**^ | .156 | .421^**^ | .208 | .302^*^ | 1 |  |  |  |  |  |  |  |  |  |  |  |  |
| Vulnerability (7) | .775^**^ | .358^**^ | .289^*^ | .520^**^ | .388^**^ | .504^**^ | 1 |  |  |  |  |  |  |  |  |  |  |  |
| **Extraversion (domain - 8)** | -.275^*^ | -.162 | -.139 | -.292^*^ | -.349^**^ | .095 | -.291^*^ | 1 |  |  |  |  |  |  |  |  |  |  |
| Warmth - 9 | -.236^*^ | -.060 | -.475^**^ | -.083 | -.319^**^ | .058 | -.113 | .704^**^ | 1 |  |  |  |  |  |  |  |  |  |
| Gregariousness - 10 | -.211 | -.169 | -.288^*^ | -.092 | -.213 | -.044 | -.074 | .703^**^ | .557^**^ | 1 |  |  |  |  |  |  |  |  |
| Assertiveness - 11 | -.282^*^ | -.142 | .161 | -.366^**^ | -.257^*^ | -.074 | -.440^**^ | .553^**^ | .197 | .174 | 1 |  |  |  |  |  |  |  |
| Activity - 12 | -.290^*^ | -.135 | -.051 | -.316^**^ | -.282^*^ | -.033 | -.351^**^ | .754^**^ | .407^**^ | .440^**^ | .431^**^ | 1 |  |  |  |  |  |  |
| Excitement-seeking - 13 | -.031 | -.028 | .131 | -.101 | -.131 | .175 | -.186 | .630^**^ | .169 | .261^*^ | .319^**^ | .363^**^ | 1 |  |  |  |  |  |
| Positive emotions - 14 | -.034 | -.102 | -.051 | -.214 | -.191 | .329^**^ | .036 | .594^**^ | .515^**^ | .279^*^ | .028 | .352^**^ | .290^*^ | 1 |  |  |  |  |
| **Openness (domain - 15)** | .080 | .284^*^ | -.059 | .187 | -.084 | .081 | -.077 | .250^*^ | .238^*^ | -.124 | .131 | .218 | .282^*^ | .286^*^ | 1 |  |  |  |
| Fantasy - 16 | .223 | .105 | -.030 | .173 | -.018 | .344^**^ | .272^*^ | .157 | .208 | -.032 | -.141 | -.003 | .231 | .406^**^ | .552^**^ | 1 |  |  |
| Aesthetics - 17 | .145 | .332^**^ | -.069 | .353^**^ | .048 | -.029 | -.023 | .032 | .111 | -.099 | -.034 | -.005 | .132 | .029 | .792^**^ | .313^**^ | 1 |  |
| Feelings - 18 | .036 | .177 | -.119 | -.066 | -.127 | .304^*^ | -.045 | .399^**^ | .437^**^ | .166 | .185 | .270^*^ | .142 | .434^**^ | .548^**^ | .231 | .470^**^ | 1 |
| Action - 19 | -.092 | .070 | .073 | .021 | -.094 | -.123 | -.287^*^ | .181 | .028 | -.134 | .318^**^ | .285^*^ | .238^*^ | -.023 | .415^**^ | -.052 | .205 | -.074 |
| Ideas - 20 | .059 | .296^*^ | .076 | .129 | .088 | -.115 | -.176 | .076 | -.032 | -.233 | .151 | .245^*^ | .245^*^ | -.079 | .686^**^ | .153 | .530^**^ | .138 |
| Values - 21 | -.179 | -.158 | -.181 | -.087 | -.254^*^ | -.110 | .037 | -.028 | .059 | -.009 | -.072 | -.130 | -.159 | .252^*^ | .125 | .096 | -.118 | .041 |
| **Agreeableness (domain - 22)** | -.010 | .101 | -.512^**^ | .343^**^ | .066 | -.119 | .082 | -.002 | .380^**^ | .042 | -.192 | -.162 | -.078 | .032 | .310^**^ | .198 | .349^**^ | .377^**^ |
| Trust - 23 | -.120 | -.038 | -.399^**^ | .005 | .083 | -.015 | -.111 | .206 | .345^**^ | .099 | .093 | .004 | .152 | .135 | .205 | -.036 | .184 | .360^**^ |
| Straightforwardness - 24 | -.103 | -.017 | -.468^**^ | .174 | .077 | -.166 | -.006 | -.115 | .277^*^ | -.020 | -.257^*^ | -.154 | -.237^*^ | -.017 | .185 | .070 | .267^*^ | .272^*^ |
| Altruism - 25 | .123 | .182 | -.088 | .030 | .069 | .295^*^ | .000 | .150 | .200 | -.061 | .066 | -.037 | .265^*^ | .172 | .275^*^ | .334^**^ | .126 | .376^**^ |
| Compliance - 26 | -.109 | .047 | -.454^**^ | .116 | -.014 | -.209 | .080 | .079 | .312^**^ | .184 | -.206 | .049 | -.106 | .109 | .202 | .209 | .199 | .203 |
| Modesty - 27 | .176 | .064 | -.094 | .522^**^ | .004 | -.141 | .329^**^ | -.452^**^ | -.126 | -.172 | -.431^**^ | -.477^**^ | -.331^**^ | -.242^*^ | .037 | .130 | .178 | -.230 |
| Tender-mindedness - 28 | -.028 | .124 | -.219 | .166 | .013 | -.066 | -.112 | .296^*^ | .360^**^ | .158 | .233 | .211 | .149 | .060 | .191 | -.006 | .201 | .444^**^ |
| **Conscientiousness (domain - 29)** | -.358^**^ | .037 | -.228 | -.233 | -.014 | -.342^**^ | -.576^**^ | .310^**^ | .238^*^ | .159 | .448^**^ | .325^**^ | .154 | -.130 | .229 | -.226 | .259^*^ | .241^*^ |
| Competence - 30 | -.468^**^ | -.141 | -.232 | -.371^**^ | -.209 | -.296^*^ | -.588^**^ | .394^**^ | .296^*^ | .217 | .381^**^ | .351^**^ | .219 | .080 | .273^*^ | -.087 | .227 | .392^**^ |
| Order - 31 | .205 | .268^*^ | .254^*^ | .102 | .119 | .145 | -.034 | .198 | .089 | .150 | .176 | .153 | .167 | .023 | -.010 | -.089 | .103 | .074 |
| Dutifulness - 32 | -.215 | -.083 | -.185 | -.191 | .092 | -.063 | -.396^**^ | .200 | .178 | .069 | .276^*^ | .211 | .128 | -.092 | .089 | -.103 | .109 | .177 |
| Achievement - 33 | -.195 | .052 | -.106 | -.236^*^ | .030 | -.072 | -.401^**^ | .402^**^ | .287^*^ | .133 | .470^**^ | .460^**^ | .200 | .034 | .274^*^ | -.120 | .143 | .258^*^ |
| Self-discipline - 34 | -.319^**^ | .035 | -.244^*^ | -.165 | -.025 | -.333^**^ | -.481^**^ | .289^*^ | .161 | .132 | .403^**^ | .356^**^ | .148 | -.083 | .389^**^ | -.104 | .351^**^ | .211 |
| Deliberation - 35 | -.420^**^ | .028 | -.355^**^ | -.111 | -.036 | -.653^**^ | -.470^**^ | -.111 | .017 | -.003 | .177 | -.113 | -.143 | -.420^**^ | -.054 | -.382^**^ | .142 | -.051 |

* p < .05; ** p < .01

Table B (continued)

|  | 19 | 20 | 21 | **22** | 23 | 24 | 25 | 26 | 27 | 28 | **29** | 30 | 31 | 32 | 33 | 34 | 35 |
| --- | --- | --- | --- | --- | --- | --- | --- | --- | --- | --- | --- | --- | --- | --- | --- | --- | --- |
| Action - 19 | 1 |  |  |  |  |  |  |  |  |  |  |  |  |  |  |  |  |
| Ideas - 20 | .329^**^ | 1 |  |  |  |  |  |  |  |  |  |  |  |  |  |  |  |
| Values - 21 | -.188 | -.162 | 1 |  |  |  |  |  |  |  |  |  |  |  |  |  |  |
| **Agreeableness (domain - 22)** | -.073 | .063 | .101 | 1 |  |  |  |  |  |  |  |  |  |  |  |  |  |
| Trust - 23 | -.119 | .114 | .212 | .631^**^ | 1 |  |  |  |  |  |  |  |  |  |  |  |  |
| Straightforwardness - 24 | -.037 | -.009 | .048 | .793^**^ | .439^**^ | 1 |  |  |  |  |  |  |  |  |  |  |  |
| Altruism - 25 | -.194 | .137 | .132 | .403^**^ | .434^**^ | .098 | 1 |  |  |  |  |  |  |  |  |  |  |
| Compliance - 26 | .068 | .033 | -.090 | .581^**^ | .215 | .374^**^ | -.014 | 1 |  |  |  |  |  |  |  |  |  |
| Modesty - 27 | -.006 | -.079 | .128 | .335^**^ | -.274^*^ | .276^*^ | -.171 | .139 | 1 |  |  |  |  |  |  |  |  |
| Tender-mindedness - 28 | .010 | .068 | -.108 | .623^**^ | .479^**^ | .433^**^ | .264^*^ | .221 | -.171 | 1 |  |  |  |  |  |  |  |
| **Conscientiousness (domain - 29)** | .308^**^ | .237^*^ | -.140 | .087 | .257^*^ | .130 | .113 | -.034 | -.347^**^ | .304^*^ | 1 |  |  |  |  |  |  |
| Competence - 30 | .072 | .212 | .072 | .044 | .272^*^ | .126 | .139 | -.101 | -.430^**^ | .299^*^ | .763^**^ | 1 |  |  |  |  |  |
| Order - 31 | .122 | .035 | -.363^**^ | -.129 | -.109 | -.080 | .002 | -.175 | -.090 | .045 | .338^**^ | .042 | 1 |  |  |  |  |
| Dutifulness - 32 | .171 | .046 | -.142 | .162 | .335^**^ | .113 | .260^*^ | -.037 | -.295^*^ | .312^**^ | .649^**^ | .449^**^ | -.009 | 1 |  |  |  |
| Achievement - 33 | .423^**^ | .238^*^ | -.103 | -.014 | .249^*^ | .014 | .187 | -.101 | -.461^**^ | .242^*^ | .824^**^ | .558^**^ | .234 | .588^**^ | 1 |  |  |
| Self-discipline - 34 | .413^**^ | .358^**^ | -.045 | .149 | .245^*^ | .166 | .097 | .105 | -.251^*^ | .237^*^ | .853^**^ | .589^**^ | .202 | .434^**^ | .707^**^ | 1 |  |
| Deliberation - 35 | .072 | .077 | -.059 | .133 | .097 | .177 | -.141 | .104 | .021 | .157 | .716^**^ | .525^**^ | .056 | .336^**^ | .350^**^ | .556^**^ | 1 |

* p < .05; ** p < .01

Table C

Intra-class correlations between twins on domains and facets of the NEO-PI-R

|  | r_mz_ | r_dz_ |
| --- | --- | --- |
| **Neuroticism (trait )** | .419** | .302** |
| Anxiety | .119 | .160 |
| Hostility | .328** | .061 |
| Depression | .337** | .325** |
| Self-conscientiousness | .345** | .351** |
| Impulsiveness | .343** | .023 |
| Vulnerability | .499** | .184 |
| **Extraversion (trait)** | .587** | .324** |
| Warmth | .436** | .467** |
| Gregariousness | .512** | .237* |
| Assertiveness | .507** | .187 |
| Activity | .541** | .189 |
| Excitement-seeking | .592** | .286** |
| Positive emotions | .325** | -.060 |
| **Openness (trait)** | .501** | .253** |
| Fantasy | .407** | .199* |
| Aesthetics | .496** | .185 |
| Feelings | .432** | .021 |
| Action | .541** | .169 |
| Ideas | .445** | .278** |
| Values | .369** | .213* |
| **Agreeableness (trait)** | .527** | .230* |
| Trust | .397** | .240* |
| Straightforwardness | .472** | .085 |
| Altruism | .378** | .224* |
| Compliance | .409** | .103 |
| Modesty | .543** | .259** |
| Tender-mindedness | .357** | .120 |
| **Conscientiousness (trait)** | .618** | .384** |
| Competence | .496** | .153 |
| Order | .359** | .290** |
| Dutifulness | .399** | .362** |
| Achievement | .549** | .219* |
| Self-discipline | .572** | .181 |
| Deliberation | .563** | .163 |

*Notes*. r_mz_ – correlations between monozygotic twin pairs; r_dz_ – correlations between dizygotic twin pairs; * p < .05; ** p < .01.

Table D
*Main effects of COMT alleles on CpG*

|  | COMT Allele | N | Mean Rank | Z/p |
| --- | --- | --- | --- | --- |
| CpG1 | MET | 23 | 33.98 | -.438 |
|  | VAL | 47 | 36.24 | .662 |
| CpG2 | MET | 23 | 35.00 | -.144 |
|  | VAL | 47 | 35.74 | .886 |
| CpG3 | MET | 23 | 33.67 | -.525 |
|  | VAL | 47 | 36.39 | .599 |
| CpG4 | MET | 23 | 32.76 | -.788 |
|  | VAL | 47 | 36.84 | .431 |
| CpG5 | MET | 23 | 31.43 | -1.169 |
|  | VAL | 47 | 37.49 | .242 |
| Mean CpG | MET | 23 | 33.43 | -.594 |
|  | VAL | 47 | 36.51 | .553 |

Table E

*Association between COMT DNAm levels and NEO-PI-R domains and facets (“greater” alternative)*

| Domain/Facet | CpG1 | | CpG2 | | CpG3 | | CpG4 | | CpG5 | |
| --- | --- | --- | --- | --- | --- | --- | --- | --- | --- | --- |
|  | BT | p | BT | p | BT | p | BT | p | BT | p |
| Neuroticism (D) | -0.524 | 1.000 | 0.364 | 0.388 | -0.277 | 1.000 | 0.784 | 0.299 | -1.148 | 1.000 |
| Anxiety | 0.828 | 0.330 | 0.411 | 0.370 | 1.690 | 0.062 | 1.394 | 0.11 | 0.277 | 0.440 |
| Hostility | -0.174 | 1.000 | -1.012 | 1.000 | -0.552 | 1.000 | -0.195 | 1.000 | -1.513 | 1.000 |
| Depression | -0.503 | 1.000 | 1.012 | 0.240 | 0.552 | 0.356 | -0.488 | 1.000 | -1.203 | 1.000 |
| Self-Consciousness | 0.155 | 0.480 | 0.364 | 0.388 | 0.416 | 0.392 | 0.098 | 0.525 | -1.148 | 1.000 |
| Impulsiveness | 0.122 | 0.482 | 0.864 | 0.243 | -2.035 | 1.000 | -0.103 | 1.000 | -0.368 | 1.000 |
| Vulnerability | -1.513 | 1.000 | -0.733 | 1.000 | 0.277 | 0.442 | 0.588 | 0.325 | -0.895 | 1.000 |
| Extraversion (D) | 0.488 | 0.342 | 0.733 | 0.314 | 0.000 | 1.000 | -0.296 | 1.000 | -2.155 | 1.000 |
| Warmth | 0.195 | 0.481 | **2.035** | **0.030** | 0.000 | 1.000 | -1.085 | 1.000 | 0.784 | 0.306 |
| Gregariousness | **1.855** | **0.036** | 1.109 | 0.199 | 0.697 | 0.326 | -0.296 | 1.000 | -0.784 | 1.000 |
| Assertiveness | 0.879 | 0.211 | 1.343 | 0.102 | 0.697 | 0.326 | 0.296 | 0.432 | -0.588 | 1.000 |
| Activity | 1.179 | 0.145 | 1.374 | 0.114 | 1.519 | 0.081 | -0.195 | 1.000 | -0.155 | 1.000 |
| Excitement-Seeking | -1.179 | 1.000 | -1.053 | 1.000 | -0.138 | 1.000 | -0.488 | 1.000 | -2.56 | 1.000 |
| Positive Emotions | -0.138 | 1.000 | 1.331 | 0.106 | -0.986 | 1.000 | -1.394 | 1.000 | -2.355 | 1.000 |
| Openness to Experience (D) | 0.524 | 0.327 | 1.343 | 0.102 | 0.970 | 0.231 | -2.155 | 1.000 | -0.895 | 1.000 |
| Fantasy | -1.179 | 1.000 | 0.324 | 0.419 | 0.552 | 0.356 | -1.172 | 1.000 | -1.203 | 1.000 |
| Aesthetics | -0.828 | 1.000 | -1.497 | 1.000 | -0.282 | 1.000 | -2.092 | 1.000 | -0.277 | 1.000 |
| Feelings | -0.503 | 1.000 | 1.374 | 0.114 | 1.243 | 0.125 | -1.172 | 1.000 | 0.834 | 0.274 |
| Actions | 0.879 | 0.211 | 0.986 | 0.199 | 0.697 | 0.326 | 0.296 | 0.432 | 0.098 | 0.492 |
| Ideas | 0.174 | 0.477 | 0.324 | 0.419 | 0.552 | 0.356 | -1.172 | 1.000 | -1.203 | 1.000 |
| Values | 1.513 | 0.090 | 1.425 | 0.088 | **1.801** | **0.045** | 1.469 | 0.090 | 1.576 | 0.088 |
| Agreeableness (D) | -1.179 | 1.000 | 0.000 | 1.000 | -0.828 | 1.000 | -2.538 | 1.000 | -0.524 | 1.000 |
| Trust | 0.220 | 0.433 | **2.745** | **0.004** | 1.571 | 0.084 | 0.404 | 0.388 | 0.742 | 0.356 |
| Straightforwardness | -0.851 | 1.000 | -0.324 | 1.000 | -1.933 | 1.000 | -2.929 | 1.000 | -0.834 | 1.000 |
| Altruism | 0.834 | 0.299 | 0.733 | 0.314 | 1.108 | 0.158 | -0.588 | 1.000 | -1.148 | 1.000 |
| Compliance | -0.503 | 1.000 | -0.364 | 1.000 | 0.552 | 0.356 | 0.195 | 0.468 | 0.834 | 0.274 |
| Modesty | 0.503 | 0.368 | -0.324 | 1.000 | -1.243 | 1.000 | 0.488 | 0.368 | **1.882** | **0.035** |
| Tender-Mindedness | -1.519 | 1.000 | -0.083 | 1.000 | 0.423 | 0.368 | -1.394 | 1.000 | -1.662 | 1.000 |
| Conscientiousness (D) | 1.172 | 0.210 | 0.733 | 0.314 | -0.697 | 1.000 | -1.676 | 1.000 | -0.784 | 1.000 |
| Competence | 0.524 | 0.327 | 1.701 | 0.054 | -0.416 | 1.000 | -1.469 | 1.000 | -0.214 | 1.000 |
| Order | 0.195 | 0.481 | -0.733 | 1.000 | -0.697 | 1.000 | -1.085 | 1.000 | -1.959 | 1.000 |
| Dutifulness | 0.828 | 0.330 | -0.287 | 1.000 | -1.127 | 1.000 | -2.092 | 1.000 | -1.108 | 1.000 |
| Achievement Striving | 0.851 | 0.274 | 1.012 | 0.240 | 0.552 | 0.356 | -1.172 | 1.000 | -1.203 | 1.000 |
| Self-Discipline | 0.503 | 0.368 | 0.687 | 0.304 | -1.243 | 1.000 | 0.488 | 0.368 | -0.155 | 1.000 |
| Deliberation | 0.250 | 0.463 | 0.222 | 0.435 | 1.041 | 0.169 | -0.210 | 1.000 | 0.710 | 0.263 |

*Notes*: BT – Barnard’s test, p – p-value for the test. Significant associations are bolded.

Table F

*Association between COMT DNAm levels and NEO-PI-R domains and facets (“less” alternative)*

| Domain/Facet | CpG1 | | CpG2 | | CpG3 | | CpG4 | | Cpg5 | |
| --- | --- | --- | --- | --- | --- | --- | --- | --- | --- | --- |
|  | BT | p | BT | p | BT | p | BT |  | BT | p |
| Neuroticism (D) | -0.195 | 0.481 | 0.733 | 1.000 | 0.0 | 1.000 | 1.085 | 1.000 | -0.784 | 0.306 |
| Anxiety | 1.207 | 1.000 | 0.864 | 1.000 | 2.676 | 1.000 | 1.682 | 1.000 | 1.483 | 1.000 |
| Hostility | -0.552 | 0.355 | **-2.038** | **0.028** | **-1.831** | **0.047** | -0.697 | 0.330 | **-2.493** | **0.008** |
| Depression | -0.195 | 0.481 | 0.733 | 1.000 | 0.000 | 1.000 | -0.296 | 0.432 | **-2.155** | **0.018** |
| Self-Consciousness | -0.220 | 0.433 | -0.624 | 0.346 | -0.857 | 0.238 | 0.303 | 1.000 | -1.445 | 0.084 |
| Impulsiveness | -0.287 | 0.481 | 0.619 | 1.000 | **-2.053** | **0.031** | -0.67 | 0.306 | 0.089 | 1.000 |
| Vulnerability | -1.179 | 0.145 | -0.364 | 0.388 | -0.138 | 0.492 | 0.879 | 1.000 | -0.524 | 0.356 |
| Extraversion (D) | 0.488 | 1.000 | 0.733 | 1.000 | 0.000 | 1.000 | -0.296 | 0.432 | **-2.155** | **0.018** |
| Warmth | 1.172 | 1.000 | 3.203 | 1.000 | 0.697 | 1.000 | 0.394 | 1.000 | 1.959 | 1.000 |
| Gregariousness | 1.180 | 1.000 | 0.465 | 1.000 | 0.571 | 1.000 | -0.404 | 0.388 | -0.742 | 0.356 |
| Assertiveness | 1.179 | 1.000 | 1.741 | 1.000 | 0.138 | 1.000 | -0.195 | 0.468 | -0.834 | 0.274 |
| Activity | 1.180 | 1.000 | 0.790 | 1.000 | -0.143 | 0.469 | -0.404 | 0.388 | -0.742 | 0.356 |
| Excitement-Seeking | -0.879 | 0.211 | -1.343 | 0.102 | 0.000 | 1.000 | -0.296 | 0.432 | **-2.155** | **0.018** |
| Positive Emotions | 0.851 | 1.000 | 1.701 | 1.000 | -0.828 | 0.274 | -0.488 | 0.368 | **-1.882** | **0.035** |
| Openness to Experience (D) | -0.524 | 0.327 | 1.053 | 1.000 | 0.416 | 1.000 | **-2.645** | **0.005** | -1.148 | 0.148 |
| Fantasy | -1.243 | 0.131 | -0.287 | 0.454 | 0.282 | 1.000 | -1.394 | 0.110 | -1.108 | 0.191 |
| Aesthetics | -0.879 | 0.211 | 0.041 | 1.000 | -0.697 | 0.326 | -1.676 | 0.075 | -0.098 | 0.492 |
| Feelings | -0.195 | 0.481 | 1.425 | 1.000 | 2.092 | 1.000 | -0.986 | 0.210 | 0.588 | 1.000 |
| Actions | 0.488 | 1.000 | 0.733 | 1.000 | 0.000 | 1.000 | -0.296 | 0.432 | -0.098 | 0.492 |
| Ideas | 0.155 | 1.000 | 0.364 | 1.000 | 1.108 | 1.000 | -0.588 | 0.325 | -0.467 | 0.344 |
| Values | 0.828 | 1.000 | 1.109 | 1.000 | 1.690 | 1.000 | 1.394 | 1.000 | 0.970 | 1.000 |
| Agreeableness (D) | -1.179 | 0.145 | 0.000 | 1.000 | -0.828 | 0.274 | **-2.538** | **0.007** | -0.524 | 0.356 |
| Trust | -0.524 | 0.327 | 2.430 | 1.000 | 1.108 | 1.000 | 0.098 | 1.000 | 0.214 | 1.000 |
| Straightforwardness | -0.879 | 0.211 | -0.287 | 0.454 | **-2.092** | **0.023** | **-2.366** | **0.013** | -0.098 | 0.492 |
| Altruism | 0.122 | 1.000 | 0.465 | 1.000 | 1.599 | 1.000 | -0.103 | 0.493 | -1.082 | 0.191 |
| Compliance | -0.879 | 0.211 | 0.041 | 1.000 | 0.697 | 1.000 | 0.394 | 1.000 | 1.273 | 1.000 |
| Modesty | 1.180 | 1.000 | -0.254 | 0.423 | -0.857 | 0.238 | 0.303 | 1.000 | 0.662 | 1.000 |
| Tender-Mindedness | -1.172 | 0.210 | 0.287 | 1.000 | 0.000 | 1.000 | **-1.775** | **0.044** | **-1.959** | **0.031** |
| Conscientiousness (D) | 1.172 | 1.000 | 0.733 | 1.000 | -0.697 | 0.326 | -1.676 | 0.075 | -0.784 | 0.306 |
| Competence | 0.834 | 1.000 | 0.733 | 1.000 | -0.277 | 0.442 | **-1.959** | **0.035** | -0.467 | 0.344 |
| Order | -0.195 | 0.481 | -0.651 | 0.314 | -0.697 | 0.326 | -0.986 | 0.210 | **-2.155** | **0.018** |
| Dutifulness | 0.856 | 1.000 | 0.532 | 1.000 | 0.000 | 1.000 | -0.972 | 0.189 | 0.429 | 1.000 |
| Achievement Striving | 1.513 | 1.000 | 1.741 | 1.000 | 0.416 | 1.000 | -0.588 | 0.325 | -0.467 | 0.344 |
| Self-Discipline | 0.828 | 1.000 | 1.109 | 1.000 | -0.423 | 0.368 | -0.697 | 0.330 | -0.416 | 0.368 |
| Deliberation | -0.155 | 0.480 | -0.733 | 0.314 | 0.970 | 1.000 | -0.784 | 0.299 | 0.467 | 1.000 |

*Notes*: BT – Barnard’s test, p – p-value for the test. Significant associations are bolded.
